# Preparation of Conductive Polyester Fibers Using Continuous Two-Step Plating Silver

**DOI:** 10.3390/ma11102033

**Published:** 2018-10-19

**Authors:** Changchun Liu, Xuelian Li, Xiaoqiang Li, Tianze Xu, Chunyu Song, Kenji Ogino, Zhijie Gu

**Affiliations:** 1 Graduate School of Bio-Applications and Systems Engineering, Tokyo University of Agriculture and Technology, Koganei, Tokyo 184-8588, Japan; s167438z@st.go.tuat.ac.jp; 2 College of Chemistry and Materials Engineering, Changzhou Vocational Institute of Engineering, Changzhou 213164, China; czlxl2015@163.com; 3 College of Textile and Clothing, Jiangnan University, Wuxi 214122, China; lixiaoqiang@jiangnan.edu.cn (X.L.); lanneret1@yahoo.com (C.S.); 4 School of Materials Science and Engineering, Tianjin University, Tianjin 300350, China; william0718@126.com

**Keywords:** continuous two-step silver plating, electroplating, electroless plating, polyester fiber, dynamic condition

## Abstract

Polyester fibers are used in various fields, due to their excellent mechanical and chemical stability. However, the lack of conductivity limits their application potential. In order to prepare conductive polyester fibers, silver is one of the most widely used materials to coat the surface of the fibers. This work aimed to prepare silver-coated polyester fibers by a continuous two-step method, which combined the operations of continuous electroless plating and electroplating. Meanwhile, we designed specialized equipment for the continuous plating of silver on the polyester fibers under a dynamic condition. The mechanical property, washability, electrical resistivity, and electrical conductivity of the resultant conductive polyester fibers obtained from different silver-plating conditions were also characterized. The results demonstrated that the conductive fibers prepared by continuous two-step silver plating equipment, had good electrical conductivity with better mechanical properties and washability.

## 1. Introduction

Polyester fibers are widely used in various fields, because of their excellent physical and mechanical properties and chemical stability. However, normal polyester fiber has a low moisture regain [1,2], higher electrical resistance [3,4], and can easily accumulate large amounts of electric charges on the surface [5,6], which results in fibers repelling each other, clothing absorbing dust and clinging onto the body, and can even produce electrical shocks or ignite flammable substances. Polyester fibers’ easily generating static electricity and poor conductivity, limit their applications in apparel and home furnishings [7,8,9]. Therefore, it is necessary to improve the electrical conductivity of the fibers. Plating a layer of silver on the surface of a polyester fiber will preserve the excellent properties of the polyester. Meanwhile, it also has the excellent electrical conductivity [10], ductility [11,12], catalytic activity [13,14], and antibacterial deodorization properties of metallic silver [15,16,17,18], and the material can also obtain a certain metallic luster and decorative effect [19,20,21,22].

The electroless silver plating method is widely used in applications to the surface metallization of fibers, due to its simple operation process, uniform coating, and controllable thickness [23,24,25,26]. However, the surface of the fiber has no catalytic activity. Therefore, using electroless plating on the surface of the fiber to achieve metal deposition requires pretreatment to impart a certain catalytic activity to the surface of the fiber [27,28]. The conventional pretreatment process mainly includes the four steps of cleaning, coarsening, sensitization, and activation, and the activation is an important step to determine the catalytic activity of the substrate [29,30,31]. The activation of the fibers often uses palladium with high reactivity, such as the sensitization–activation two-step method [32,33,34], the colloidal palladium activation method [35,36], the liquid ion palladium activation method [37,38], and so on. Due to the highly toxic and expensive nature of palladium, as well as the great potential of polluting the plating layer [39,40], these types of methods are not conducive to the preparation and application of the electroless silver-plated fibers. It has been reported that in the process of pretreatment, silver nitrate has been used by replacing the palladium compound as an activation solution for electroless plating to form a silver catalytic center on the surface of the fiber [41], while nitrate is also considered as a potentially hazardous [42]. NaClO-HCl silver activation system also proved to be a good applicability in the silver-coating industry [43]. But these activation steps all prolong the time of electroless silver-plating, which is not conducive to production. In this work, the conventional activation step in the pretreatment process was integrated into the subsequent electroless silver plating step, reducing the number of steps and using palladium-free, cyanide-free materials.

To the best of our knowledge, the study of fiber electroless silver plating has mainly focused on chemical plating under static conditions where the technology tends to be more mature [44,45,46,47,48,49], but that the study of the dynamic continuous silver plating of fibers has been seldom reported. In addition, due to the shorter reaction time, the electroless silver-plated fibers have a thin silver coating on the surface with very poor durability. Therefore, further electroplating treatment is generally required [50,51]. The first and best developed process in the electroplating industry is cyanide electroplating silver plating. The plating solution forms a coordination complex with CN^−^ and Ag^+^, which has high stability and good dispersion of the plating solution. The silver plating layer using cyanide electroplating has high bonding fastness to the substrate [52,53], but cyanide is highly toxic, so cyanide electroplating silver must be replaced [54,55,56].

This work aimed to prepare silver coated polyester fibers by a continuous two-step method, which combined the operations of continuous electroless plating and electroplating. In the electroless silver-plating step, the polyester fiber, after being pretreated by the sensitization washing, was directly immersed into the electroless plating solution for electroless plating silver. In the second step, the electroless silver-plated fiber was subjected to a cyanide-free electroplating silver treatment by using sulfosalicylic acid as the plating solution. The whole process was continuous, and specialized continuous industrial silver-plating equipment was also designed.

## 2. Experimental

### 2.1. Materials

Polyester fibers (210D/36F) were purchased from Vekstar Textile (Shanghai) Co., Ltd. (Shanghai, China). Sodium hydroxide (NaOH, ≥96.0%), potassium hydroxide (KOH, ≥85.0%), lauryl sodium sulfate (LSS, >97.0%), stannous chloride dihydrate (SnCl_2_·2H_2_O, ≥98.0%), silver nitrate (AgNO_3_, ≥99.8%), aqueous ammonia (NH_3_·H_2_O, 25.0–28.0%), glucose (C_6_H_12_O_6_, AR), sulfosalicylic acid dihydrate (≥99.0%), ammonium acetate (≥98.0%), and hydrochloric acid (36.0–38.0%) were purchased from Sinopharm Chemical Reagent Co., Ltd. (Shanghai, China) and used without further purification.

### 2.2. Deposition of Silver on Polyester Fibers by Electroless Plating under the Dynamic Condition

**Scheme 1:**
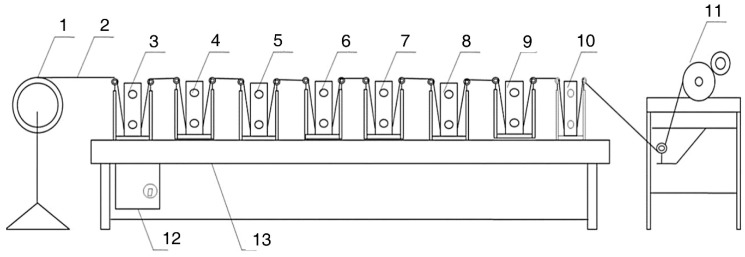
The equipment for the continuous electroless plating of silver under a dynamic condition. (1) return wire rack; (2) polyester fiber (3) cleaning tank; (4) coarsening tank; (6) sensitizing tank; (8) electroless silver-plating tank; (10) drying tank; (11) fibers winding collecting device; (12) control panel; (13) heating device; (5), (7), and (9) washing tank.

The general processes of preparing a conductive metal layer on polymeric fibers or plastics are mainly composed of two parts: (1) the pretreatment including cleaning, coarsening, sensitizing, and activating; and (2) the deposition of silver through the redox reaction. In this study, both the pretreatment and the silver deposition were continuously operated in a series of self-made reactive tanks, as shown in Scheme 1. The whole process was operated under a dynamic condition. The fiber winding collecting device at the end of the equipment provided the drafting force. The winding speed was controlled by the control panel, and the reaction time of the fibers in each plating tank could be controlled by different winding speeds and the number of windings. The size of the groove in the equipment was 13.5 cm × 8.5 cm × 14.5 cm. The lining and the drum in the tank were made of polytetrafluoroethylene (PTFE).

Polyester fibers were cleaned using a mixed solution of NaOH and lauryl sodium sulfate (LSS), followed by coarsening in NaOH solution. The sensitizing of polymeric fibers was conducted using a SnCl_2_ solution to make a Sn_2_(OH)_3_Cl gel-layer on the surface of the fibers. Then, the sensitizing fibers were put into the solution of Tollens’ reagent and glucose for the silver deposition.

### 2.3. Deposition of Silver by Continuous Electroplating

**Scheme 2:**
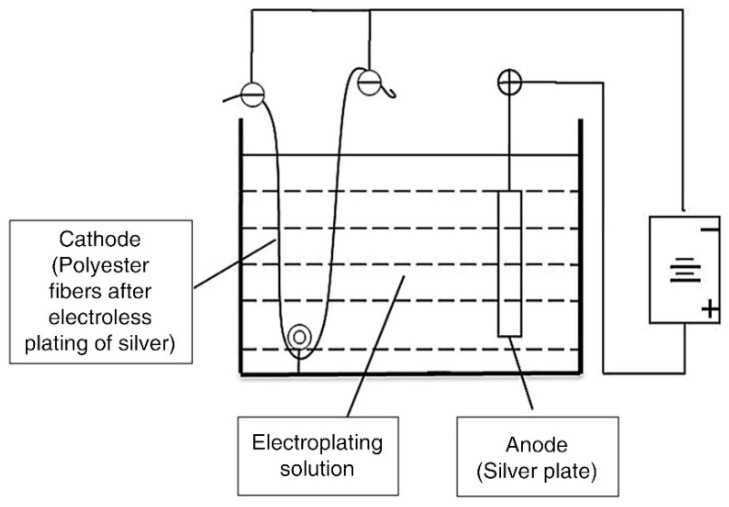
The equipment for electroplating silver on polyester fibers.

The polyester fibers with silver deposition of electroless plating were connected with a negative wire for electroplating, as shown in Scheme 2. In detail, a pure silver plate with the dimension of 80 mm × 40 mm × 5 mm was used as an anode material and a direct current with a voltage ranging from 1 V to 10 V was used as an energy resource. The polyester fibers, after being coated with a thin layer of silver by electroless plating, were directly used for electroplating. The electroplating solution was a mixture with a pH of about 9, prepared by using 120 g/L of sulfosalicylic acid, 10 g/L of potassium hydroxide, 30 g/L of silver nitrate, 50 g/L of ammonium acetate, and 60 mL/L of aqueous ammonia.

### 2.4. Characterization

Fourier-transform infrared (FTIR) spectra were obtained on a Spectrum Two IR Spectrometer (PerkinElmer, Waltham, MA, USA). The surface morphology of different fibers was examined with a Hitachi 3400N scanning electron microscope (SEM, Hitachi, Japan) at an accelerating voltage of 15 kV. XRD patterns of different fibers were measured with a D8 ADVANCE X-ray diffractometer (Bruker AXS GmbH, Karlsruhe, Germany) with a tube voltage of 40 KV, tube current of 1100 mA, a scanning range from 3 to 90 degrees, and scanning speed of 4 degrees per minute. The breaking strength and elongation at the break of the fibers were characterized by a YG020B Electronic Single Yarn Strength Tester (Changzhou No. 2 Textile Machine Co., Ltd., Changzhou, China) with a clamping length of 250 mm and a pre-tension of 10 CN. The resistivity of the conductive polyester fibers was measured with a Fluke 175C True RMS Digital Multimeter Shenzhen Xu Da Century Technology Co., Ltd. (F175C, Shenzhen, China). Ten readings at different locations of the fibers at a distance of 10 cm were taken randomly and the average was recorded.

## 3. Results and Discussion

### 3.1. Mechanism Discussion of Electroless Plating under the Dynamic Condition

#### 3.1.1. Pretreatment on Polyester Fibers

The processes of pretreatment on polyester fibers for electroless plating have been thoroughly investigated in previous studies [44,57,58,59,60]. To the best of our knowledge, only three steps are needed for the pretreatment including the cleaning, coarsening, and sensitizing. The activation step can be integrated into the subsequent electroless plating step.

**Figure 1:**
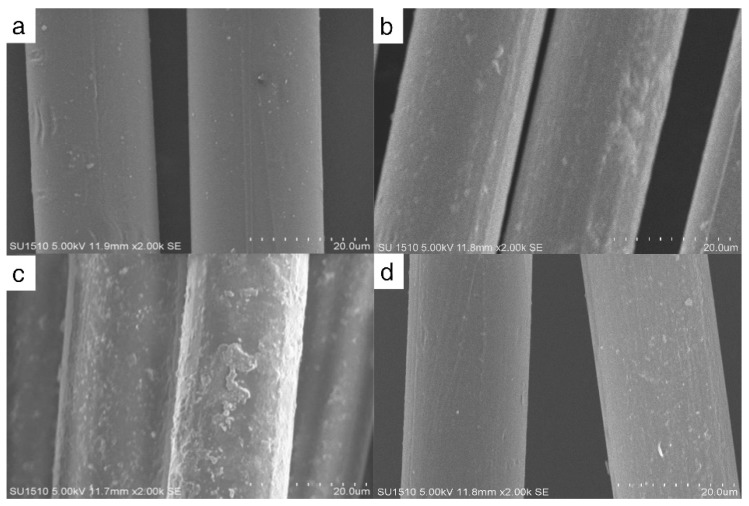
SEM images of polyester fibers: (**a**) before any treatment; (**b**) after cleaning with NaOH and lauryl sodium sulfate (LSS); (**c**) after coarsening with NaOH; and (**d**) after sensitizing with SnCl_2_.

The aim of cleaning is to remove the oil and/or other impurities that are located on the surface of the fibers in the processes of production and transportation. In this study, we used a mixture of 0.07 g/mL NaOH and 0.002 g/mL LSS to clean the surface of the polyester fibers. Both the NaOH and LSS could remove the oil from the surface of the polyester fibers, and the LSS also could serve as the surfactant to reduce the surface tension of the polyester fibers. The SEM image of the polyester fibers after cleaning is shown in Figure 1b.

The second step of pretreatment was coarsening. Coarsening is done to increase the roughness of the surface of the fibers by chemical etching and the chemical reaction between the plating solution and fibers to form uniform pores and grooves on the surface, thus forming the “locking effect” and enhancing the hydrophilicity of the substrate [28]. Therefore, the bonding strength between the coating and the substrate will be improved. In this work, the surface of the fibers was chemically etched by the NaOH solution, which should have an appropriate concentration. Too high a concentration will cause serious damage to the fiber surface and a high loss rate of the fiber mass, greatly reducing the mechanical properties of the fiber, while too low a concentration cannot meet the need of coarsening. In this study, the coarsening condition was that the fibers were coarsened in 100 g/L NaOH solution at 80 °C for 5 min. Figure 1c shows the SEM image of the coarsened polyester fibers where there were more pits and holes on the surface of the fiber, and the roughness obviously increased.

The next step of pretreatment was sensitizing. In this work, a mixed solution of 30 g/L of SnCl_2_ and 40 mL/L of hydrochloric acid was used as the sensitizing solution. After the fibers were immersed in the sensitizing solution, Sn_2_(OH)_3_Cl thin films with a slightly soluble gel were formed by the hydrolysis of stannous chloride during subsequent water washing [61]. This gel-like substance was adsorbed on the fiber surface to ensure the uniform adsorption of divalent tin ions onto the gel’s surface, which facilitated the uniform deposition of silver particles during electroless silver plating. Therefore, sensitization mainly occurs in the water washing process, and the reaction process is shown in Equations (1) and (2). Sn(OH)Cl and Sn(OH)_2_ combine to form a slightly soluble gelatinous substance, Sn_2_(OH)_3_Cl.

SnCl_2_ + 2H_2_O → Sn(OH)_2_ + HCl (1)

SnCl_2_ + 2H_2_O → Sn(OH)Cl + HCl (2)

Figure 1d shows the SEM image of the sensitized polyester fiber surface, showing that the polyester fiber surface was obviously covered with a more uniform and continuous gel-like film.

#### 3.1.2. Electroless Silver Plating on Polyester Fibers

In the electroless plating solution, Ag(NH_3_)_2_^+^ is first reduced by Sn^2+^ adsorbed on the surface of the fiber to form metal Ag particles, the reaction formula of which is shown in Equation (3). Thus, the Ag particles are deposited on the surface of the fiber where the stannous ion is dispersed. The Ag particles have strong catalytic activity and become the catalytic center of the electroless silver plating reaction. Similarly, [Ag(NH_3_)_2_]^+^ in the silver ammonia solution is reduced to metal Ag particles with glucose, and deposited on the surface of the fiber around the catalytic center to obtain a metallic silver layer, the reaction formula of which is shown in Equation (4). Therefore, in this non-activated direct electroless silver plating process, the sensitized-washed fiber will first form Ag particles on the surface of the fiber as the catalytic center of the whole reaction, so that the surface of the fiber has catalytic activity.

2[Ag(NH_3_)_2_]^+^ + Sn^2+^→ Sn^4+^ + 2Ag↓ + 4NH_3_ (3)

C_6_H_12_O_6_ + 2[Ag(NH_3_)_2_]OH → RCOONH_4_ + 3NH_3_ + 2Ag↓ + H_2_O (4)

#### 3.1.3. Influence of the Transmission Condition on the Continuous Electroless Plating Silver

**Figure 2:**
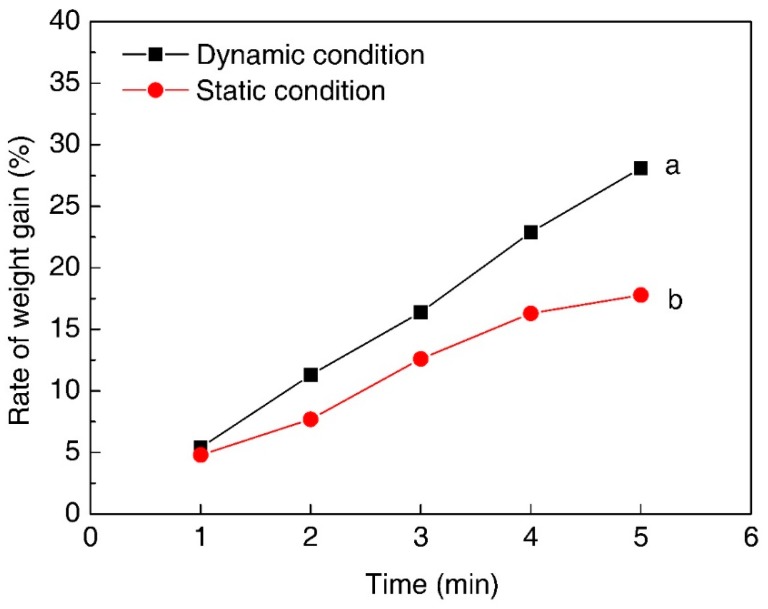
Deposition rate of silver particles under (a) dynamic, and (b) static conditions.

Continuous silver plating on fibers under a dynamic condition can facilitate deposition efficiency, which is significantly different from that under a static condition. Under the same pretreatment conditions, we performed silver plating contrast experiments in a static beaker under a dynamic condition and obtained the contrast chart of the deposition rate of silver particles under dynamic and static conditions at different plating times (Figure 2). This demonstrated that the weight gain of the fibers under the static condition was obviously lower than that under the dynamic condition, and the deposition rate became lower and lower with the increase of plating time, while the deposition rate of silver particles under the dynamic condition was higher and had less influence on time. The self-made test equipment allowed the fibers to fully make contact with the reaction liquids under the dynamic transmission, thus achieving a better silver plating effect than under the static condition, which may be because the fibers mainly reacted with the silver ammonia ion around them. When the silver was plated under a static condition, the concentration of silver ammonia ions near the fibers decreased with the progress of the reaction, so the deposition rate of the silver particles decreased continuously. However, the dynamic transmission condition facilitated the increase in the diffusion rate of silver ammonia ions onto the surface of the fibers, so the deposition rate of silver particles had little change.

### 3.2. Influence of Electroplating Process Conditions on Deposition of Silver

#### 3.2.1. Influence of Power Supply Method on Electroplating Silver

During the electroplating process, the silver layer was first deposited onto the fibers near the clamp and then extended along the radial direction of the fibers. In this process, the newly deposited silver was gradually coated onto the surface of the fibers and was closely combined with the metal silver coated by the previous electroless plating, which became the effective cathode area in the electroplating process. The new coating also reduced the fiber resistivity and increased the cathode area. In addition, due to the smaller diameter and longer length of the polyester fiber, the specific surface area of the fiber was larger, resulting in a more complex change in the cathode area of the fiber [62]. If the traditional constant current method was used to electroplate the fibers, the cathode area would change, and the cathode current density in the system would be unstable, which would cause the surface of the fibers to fluctuate greatly, which is not conducive to the formation of high-quality coatings. As such conditions are comparatively easier to control in the constant voltage method, in this study, we used a constant voltage power supply method to control the fiber electroplating experiment.

#### 3.2.2. Influence of Control Voltage on Electroplating Silver

**Figure 3:**
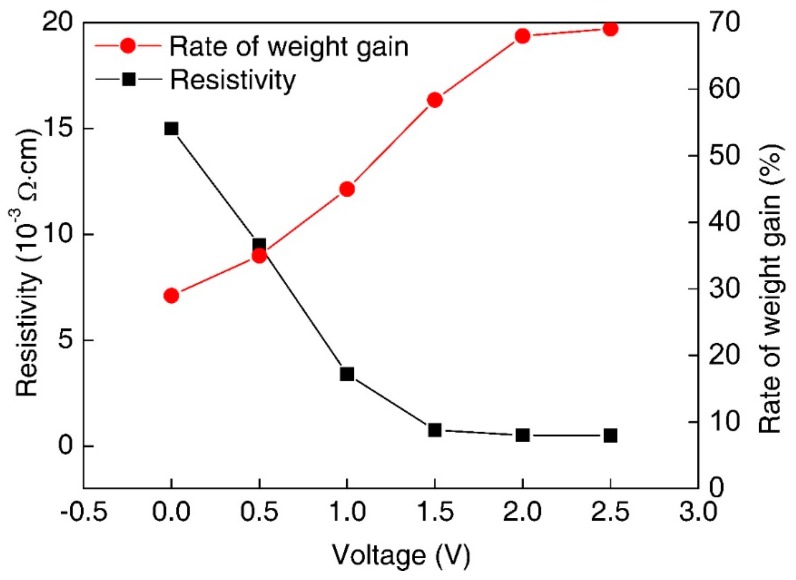
Influence of voltage on the resistivity and the rate of weight gain.

For the same electroplating time of 4 min, the influence of the plating voltage on fiber resistivity and the rate of weight gain is shown in Figure 3. With the increase in the electroplating voltage, the fiber rate of weight gain always increased, while the resistivity of the fiber gradually decreased, and after the voltage rose to above 1.5 V, the resistivity did not substantially decrease. This is because with the increase in voltage, the deposition rate of silver particles on the fiber surface was accelerated, resulting in the increased rate of weight gain of the fiber, and the silver particles deposited on the fiber surface were closely combined with the existing silver layer, resulting in the decrease of the fiber resistivity. When the voltage continues to increase, the deposition rate of silver particles will continue to increase; too fast a deposition rate will easily lead to the accumulation of particles on the silver layer, which makes the surface rough and uneven, resulting in a slow decline in resistivity. However, if the voltage is too high, it is easy to "scorch" the fibers. When the plating voltage reaches 2.6 V, the “scorch” phenomenon occurs in the fiber coating. When the voltage is lower, the deposition rate of silver particles is slow, and the silver plating efficiency is also lower. Therefore, the best control voltage range is 1.5–2.0 V.

#### 3.2.3. Influence of Electroplating Time on Electroplating Silver

**Figure 4:**
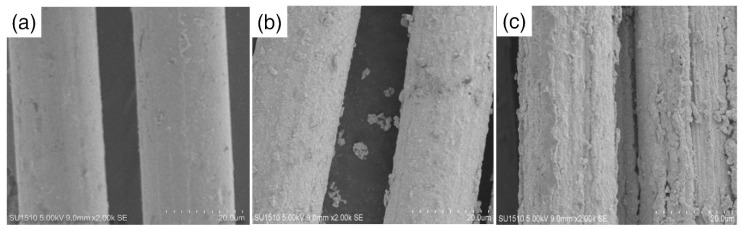
SEM images of polyester fibers for different electroplating time: (**a**) 0 min; (**b**) 4 min; (**c**) 8 min.

Figure 4 presents the SEM images of the silver-plated layer on the surface of the fiber for different plating times. The silver particles were first uniformly deposited on the surface of the fiber to form a continuous dense silver coating (Figure 4b). With prolonged electroplating time, the deposition of silver particles increased and the coating on the surface of the fiber became thicker. However, with too long a time, the deposited silver particles tended to accumulate in certain parts of the coating and caused the coating to become rougher (Figure 4c).

**Figure 5:**
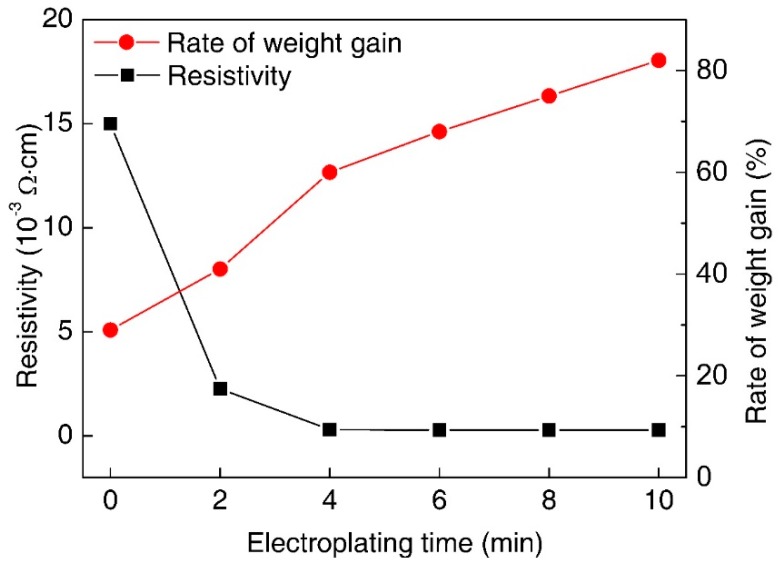
Influence of electroplating time on resistivity and the rate of weight gain.

The effects of different plating times on the resistivity and the rate of weight gain of the polyester fibers were investigated under the same plating voltage (1.6 V). The results are shown in Figure 5. With prolonged electroplating time, the rate of weight gain of the fiber increased constantly, while the resistivity decreased continuously. The resistivity remained basically unchanged after 4 min of electroplating. Different plating times can produce different coating thicknesses, but if the coating is too thick, the flexibility of the fibers will deteriorate, so in this work, the electroplating time was controlled at 4 min.

### 3.3. Silver Composition Analysis

**Figure 6:**
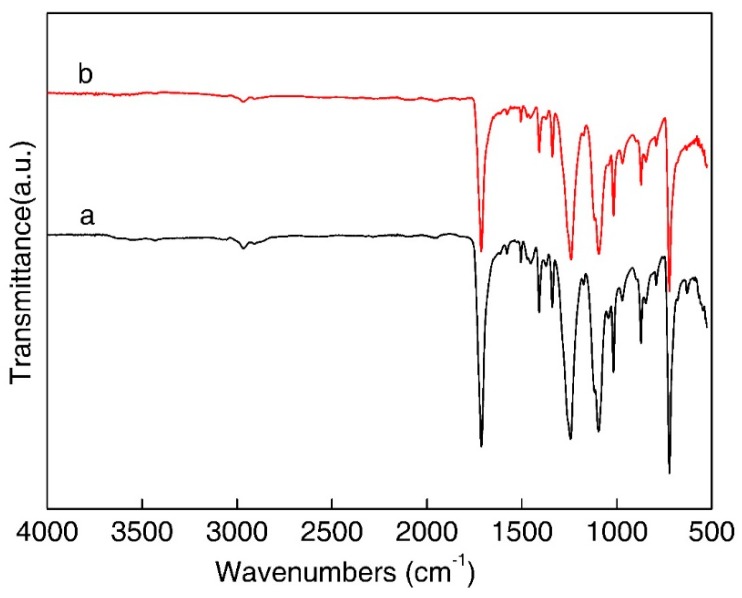
FTIR spectra of the polyester fibers (a) before and (b) after silver plating.

Figure 6 shows the infrared spectra of the polyester fibers before and after silver plating. It can be seen that the positions of the absorption peaks of the polyester fibers did not change before or after silver plating, which indicates that the basic structure of the polyester fiber is not affected by silver plating. Meanwhile, the intensity of the infrared absorption peaks of the silver-plated fiber became weaker as the surface of the fiber was coated with a dense silver layer, which reflects part of the infrared light and weakens the infrared absorption intensity of the fiber. In addition, no other new absorption peaks were observed on the spectrogram, indicating that there was no other impurity on the surface of the fiber.

**Figure 7:**
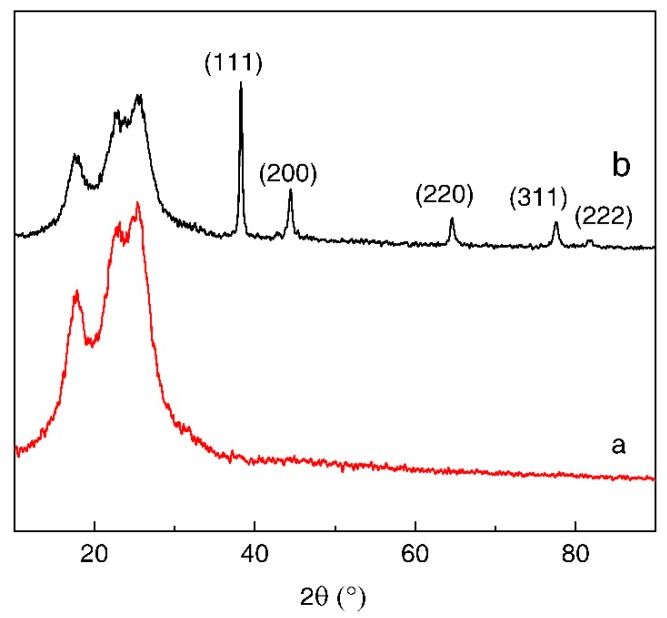
XRD patterns of the polyester fiber (a) before and (b) after silver plating.

The XRD patterns of the polyester fiber before and after silver plating are shown in Figure 7. Figure 7a demonstrates that the polyester fibers before silver plating had three diffraction peaks of polyester at 18°, 22°, and 26°, respectively, and had no other crystal diffraction peaks. Figure 7b shows that there were strong diffraction peaks at 38°, 44°, 64°, 77°, and 82°, which corresponded to the 111, 200, 220, 311, and 222 crystal plane diffraction peaks of the elemental silver crystallites, respectively. The diffraction peaks of the elemental silver were sharp and narrow, and there were no other diffraction peaks, indicating that the polyester fiber surface was coated with metallic silver with good crystallinity and high purity [63]. The characteristic diffraction peaks of the polyester fibers in Figure 7b were obviously weakened, which also indicated that the silver layer on the fiber surface was more uniform and denser.

### 3.4. Mechanical Properties Analysis

The mechanical properties of the polyester fibers at different processing stages were tested by the Electronic Single Yarn Strength Tester (YG020B, Changzhou No. 2 Textile Machine Co., Ltd., Changzhou, China). Each group of samples was tested ten times and averaged. The results were shown in Table 1.

The results show that the fiber breaking strength and the elongation at break of the coarsened polyester fiber were reduced by 12.48% and 30.63%, respectively, when compared to those of the original polyester fiber. The breaking strength and elongation at break of the fibers after electroless silver plating were 2.53% and 15.78%, higher than those before electroless plating, respectively. After electroplating, they further increased by 1.72% and 2.29%, respectively. This was because the fiber was hydrolyzed with a concentrated alkali at a high temperature during coarsening, and its surface was damaged by etching. After calculation, the mass loss rate after coarsening was about 9.8%, which led to a decrease in the mechanical property. After the electroless silver-plating, the surface of the fiber was covered with a dense silver layer. Furthermore, the metallic silver had good ductility. So, the silver layer can share part of the extra tensile force for the fiber when stretched, resulting in the increase of the breaking strength of the silver-plated fiber. The electroplated metallic silver layer completely covered the fibers and slightly enhanced the mechanical properties of the fibers. However, due to the thinner silver layer, there was less of an increase in the breaking strength of the silver-plated fiber.

### 3.5. Washing Fastness Analysis

**Figure 8:**
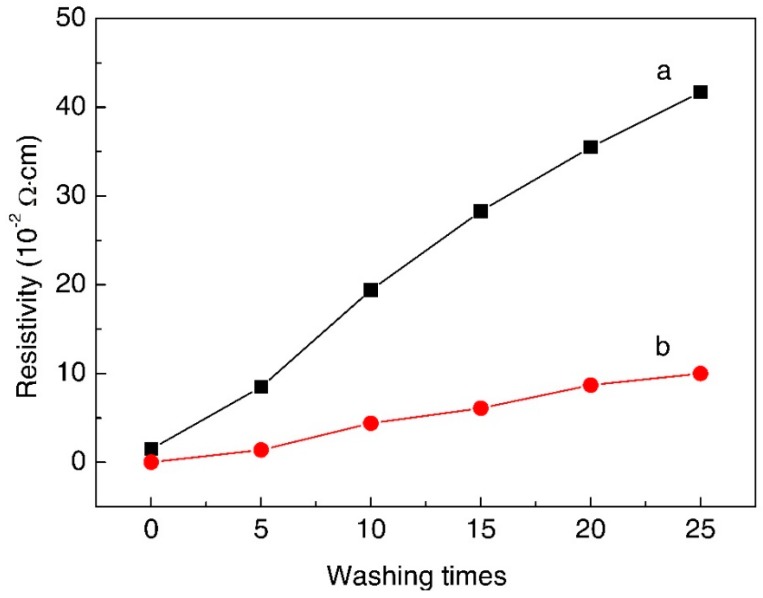
Influence of washing times on the resistivity of different fibers: (a) electroless silver-plated fiber; (b) electroplated silver-plated fiber.

**Figure 9:**
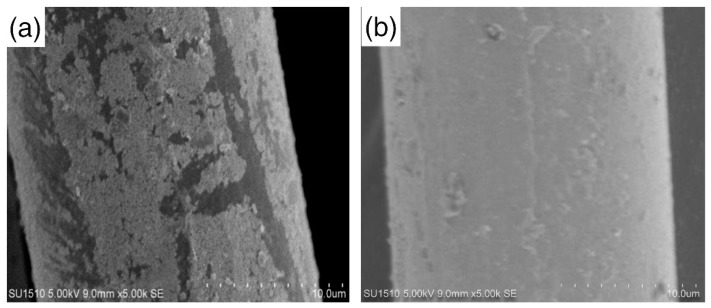
SEM images of conductive polyester fibers after being washed for 30 times: (**a**) electroless silver-plated fibers; (**b**) electroplated silver-plated fibers.

The silver layers on the electroless or electroplated silver-coated polyester fibers have a great likelihood of being removed by the act of washing. Therefore, it is necessary to examine the washing fastness. The resistivity changes and the SEM images of the electroless and electroplated silver-coated polyester fibers are shown in Figure 8 and Figure 9, respectively.

In Figure 8, the resistivities of the electroless silver-plated fiber and electroplated silver-plated fiber increased with the increase of washing times, but the increase of resistivity of the electroplated silver-plated fiber was slower than that of the electroless silver-plated fiber, indicating that the electroplated silver-plated fiber had better surface fastness and washability. This was due to the rapid deposition of silver particles on the silver surface of the electroless silver-plated fiber during the electroplating process, resulting in a thicker and denser silver layer, which improved the washability of the electroplated silver-plated fiber.

Figure 9 shows the SEM images of the electroless silver-plated fibers and the electroplated silver-plated fibers after washing 30 times. After washing, the silver layer on the surface of the electroless silver-plated fiber severely peeled off, while only some loose silver particles peeled off from the surface of the electroplated silver-plated fiber, and the main body of the silver layer was in good condition. This may be because the shorter time of continuous electroless plating silver caused a thinner coating layer and a weaker binding force between the silver layer and the fiber, while the electroplating after continuous electroless plating significantly increased the thickness of the silver layer, and the binding force between the silver layer and the fiber was also obviously enhanced.

### 3.6. Conductive Properties Analysis

**Figure 10:**
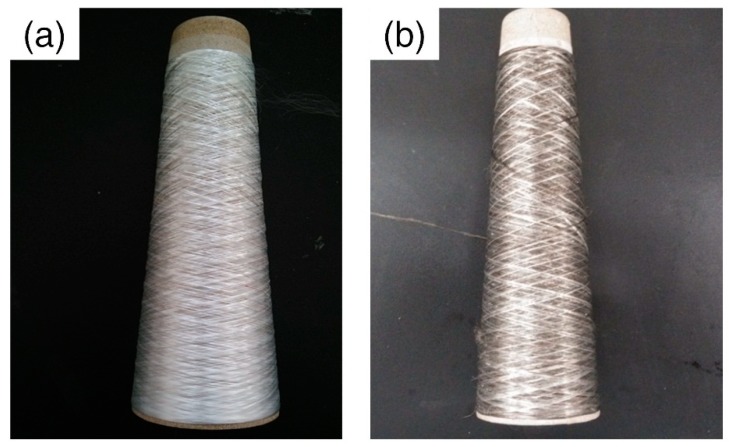
Photographs of winding bobbins of polyester fibers: (**a**) before silver plating; (**b**) after silver plating.

Figure 10b shows the polyester fiber prepared by using the self-made continuous silver plating equipment. The winding bobbin made by the continuous silver plating equipment was well-formed, and the silver-plated fiber had a silver-white metallic luster. We measured the resistivity of the polyester fibers before and after silver-plating. The resistivities of the original polyester fiber, electroless silver-plated fiber, and electroplated silver-plated fiber were 9.5 × 10^10^ Ω·cm, 1.5 × 10^−2^ Ω·cm, and 2.3 × 10^−4^ Ω·cm, respectively. The conductivity of the polyester fiber was significantly improved by silver-plating.

**Figure 11:**
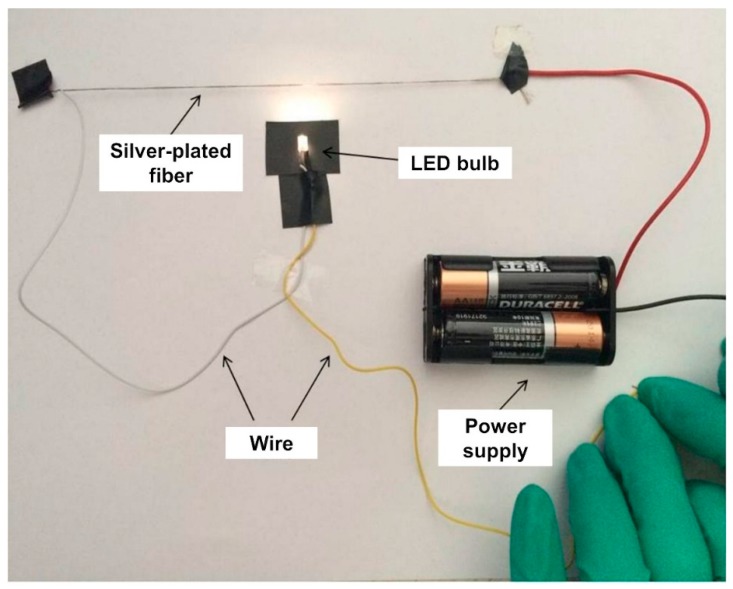
The luminescence experiment of a small bulb.

In this study, the luminescence experiment of a small bulb was also conducted. As shown in Figure 11, the two ends of the silver-plated fiber were connected to two wires and fixed on insulating paper with an insulating tape. After the wires connected the LED bulb to the power supply (3 V), the small bulb emitted an obvious bright light and the brightness was constant and stable for 10 min, which indicated that the prepared fiber surface was uniformly and continuously covered with the silver plating layer, and the fiber could conduct electrons and had good electrical conductivity.

## 4. Conclusions

In this study, a continuous two-step silver plating method was developed with polyester fibers used as the substrate material, which combined the operations of continuous electroless plating under a dynamic condition and continuous electroplating. Furthermore, we designed specialized equipment for the continuous plating of silver on the polyester fibers. According to the machine conditions, the pretreatments, electroless silver-plating, and electroplating silver plating process were improved accordingly, and the silver-plated conductive fibers were successfully prepared. The influence of the power supply method, control voltage, and electroplating time on electroplating silver was studied. The optimal conditions for electroplating silver conductive polyester fibers should include the power supply method having a constant voltage power, the best control voltage range of which is 1.5–2.0 V, and an electroplating time of 4 min. The Ag-coated polyester fibers were characterized by SEM, FTIR, and XRD. Moreover, the mechanical properties and washing fastness of the electroplating silver-plating fibers were compared with the electroless silver-plating fibers. The results demonstrated that after the continuous two-step silver plating, the surface coating of the fiber was obviously thickened, and the surface silver particles were denser and continuous, with better mechanical properties and washability. The electrical resistivity reached 2.3 × 10^−4^ Ω·cm, and the conductivity was obviously improved. The luminescence experiment of a small bulb also showed that the conductive fiber prepared by the continuous two-step silver plating method had good electrical conductivity.

## Figures and Tables

**Table 1:** Comparison of the mechanical properties of different fibers.

Sample	Breaking Strength (CN)	Elongation at Break (%)
Original polyester fiber	1041.81	23.57
Coarsening treated polyester fiber	911.23	16.35
Electroless silver-plated polyester fiber	934.25	18.81
Electroplated silver-coated polyester fiber	950.31	19.24
